# HDL-apoA-II Is Strongly Associated with 1-Year Mortality in Acute Heart Failure Patients

**DOI:** 10.3390/biomedicines10071668

**Published:** 2022-07-11

**Authors:** Iva Klobučar, Vesna Degoricija, Ines Potočnjak, Matias Trbušić, Gudrun Pregartner, Andrea Berghold, Eva Fritz-Petrin, Hansjörg Habisch, Tobias Madl, Saša Frank

**Affiliations:** 1 Department of Cardiology, Sisters of Charity University Hospital Centre, 10000 Zagreb, Croatia; iva.klobucar@gmail.com (I.K.); matias.trbusic@gmail.com (M.T.); 2 School of Medicine, University of Zagreb, 10000 Zagreb, Croatia; vesna.degoricija@mef.hr; 3 Department of Medicine, Sisters of Charity University Hospital Centre, 10000 Zagreb, Croatia; 4 Institute for Clinical Medical Research and Education, Sisters of Charity University Hospital Centre, 10000 Zagreb, Croatia; ines.potocnjak@yahoo.com; 5 Institute for Medical Informatics, Statistics und Documentation, Medical University of Graz, 8036 Graz, Austria; gudrun.pregartner@medunigraz.at (G.P.); andrea.berghold@medunigraz.at (A.B.); 6 Clinical Institute of Medical and Chemical Laboratory Diagnostics, Medical University of Graz, 8036 Graz, Austria; eva.fritz-petrin@medunigraz.at; 7 Gottfried Schatz Research Center, Molecular Biology and Biochemistry, Medical University of Graz, 8010 Graz, Austria; hansjoerg.habisch@medunigraz.at (H.H.); tobias.madl@medunigraz.at (T.M.); 8 BioTechMed-Graz, 8010 Graz, Austria

**Keywords:** acute heart failure, high-density lipoprotein, apoA-II, HDL-p, mortality, NMR spectroscopy, prognostic biomarkers, risk

## Abstract

The prognostic value of the subset of high-density lipoprotein (HDL) particles containing apolipoprotein (apo)A-II (HDL-apoA-II) in acute heart failure (AHF) remains unexplored. In this study, baseline serum levels of HDL-apoA-II (total and subfractions 1–4) were measured in 315 AHF patients using NMR spectroscopy. The mean patient age was 74.2 ± 10.5 years, 136 (43.2%) were female, 288 (91.4%) had a history of cardiomyopathy, 298 (94.6%) presented as New York Heart Association class 4, and 118 (37.5%) patients died within 1 year after hospitalization for AHF. Multivariable Cox regression analyses, adjusted for age and sex as well as other clinical and laboratory parameters associated with 1-year mortality in the univariable analyses, revealed a significant inverse association of HDL-apoA-II (hazard ratio (HR) 0.67 per 1 standard deviation (1 SD) increase, 95% confidence interval (CI) 0.47–0.94, *p* = 0.020), HDL2-apoA-II (HR 0.72 per 1 SD increase, 95% CI 0.54–0.95, *p* = 0.019), and HDL3-apoA-II (HR 0.59 per 1 SD increase, 95% CI 0.43–0.80, *p* < 0.001) with 1-year mortality. We conclude that low baseline HDL-apoA-II, HDL2-apoA-II, and HDL3-apoA-II serum levels are associated with increased 1-year mortality in AHF patients and may thus be of prognostic value in AHF.

## 1. Introduction

Despite advances in diagnostics and therapy, heart failure (HF) is one of the main causes of morbidity and mortality worldwide [1]. Acute heart failure (AHF) develops as either a de novo clinical syndrome with a rapid onset of signs and symptoms of HF or deterioration of signs and symptoms of HF in chronic heart failure (CHF) patients [2].

It is well-established that, in HF, hemodynamic impairment and thereby its associated tissue hypoperfusion and congestion, with a consequent increase in circulating natriuretic peptides and sympathetic activity as well as chronic inflammation and insulin resistance, cause a metabolic derangement highlighted by a profound catabolic dominance [3,4]. Such a metabolic constellation, accompanied by wasting as well as decreased appetite and serum lipoproteins, has been shown to be associated with a poor prognosis in HF patients [3,5,6,7].

In routine clinical laboratories, high-density lipoprotein cholesterol (HDL-C) has been used for decades as the principal clinical measure of HDL plasma levels. Although epidemiological studies established an inverse relationship between HDL-C and cardiovascular risk, clinical trials provided clear evidence that the concentration of circulating HDL particles (HDL-p) is a better predictor of CVD risk than HDL-C [8,9].

HDL constitutes a heterogeneous mixture of particles that differ in size, composition, and function [10]. While cholesteryl ester, free cholesterol, and phospholipids are principal lipid constituents of HDL, apolipoprotein (apo)A-I and A-II are the major HDL-associated apolipoproteins [11]. Immunoaffinity chromatography separates HDL into two major subpopulations—namely, those containing only apoA-I or both apoA-I and apoA-II, as well as a minor subpopulation containing only apoA-II [12,13,14].

ApoA-II confers unique structural, metabolic, and functional features to HDL, highlighted by the increased HDL particle stability, resistance to the HDL remodeling machinery, and altered cardiovascular protective activities [15,16,17,18,19,20,21,22,23,24]. Although clinical and intervention studies in mouse models of HF have established an association of HDL-C, HDL-apoA-I, and HDL-p with disease severity and outcome in CHF and AHF [25,26,27,28,29,30,31], the role and prognostic value of the subset of HDL particles containing apoA-II (HDL-apoA-II) in CHF and AHF remain unexplored. Therefore, in this study, we determined the serum levels and examined the prognostic utility of HDL-apoA-II in AHF patients, in comparison with HDL-apoA-I and HDL-p, the previously established prognostic biomarkers in AHF and CHF [28,30,31].

Here, we show that the reduced circulating HDL-apoA-II serum levels are associated with increased 1-year mortality in AHF patients, suggesting that HDL-apoA-II might be of prognostic value in AHF.

## 2. Materials and Methods

### 2.1. Study Design and Patients

This AHF-2 study was a prospective, observational, single-center research study that included consecutive adult Caucasian patients who were hospitalized due to AHF. The diagnosis and standard treatment of AHF were based on the definition and guidelines given by the European Society of Cardiology [2].

Exclusion criteria were less than 18 years of age, concomitant acute or chronic inflammatory disease, exacerbated chronic pulmonary disease, severe renal failure (serum creatinine ≥ 400 μmol/L), active malignant disease, pregnancy, and refusal to participate.

**Scheme 1:**
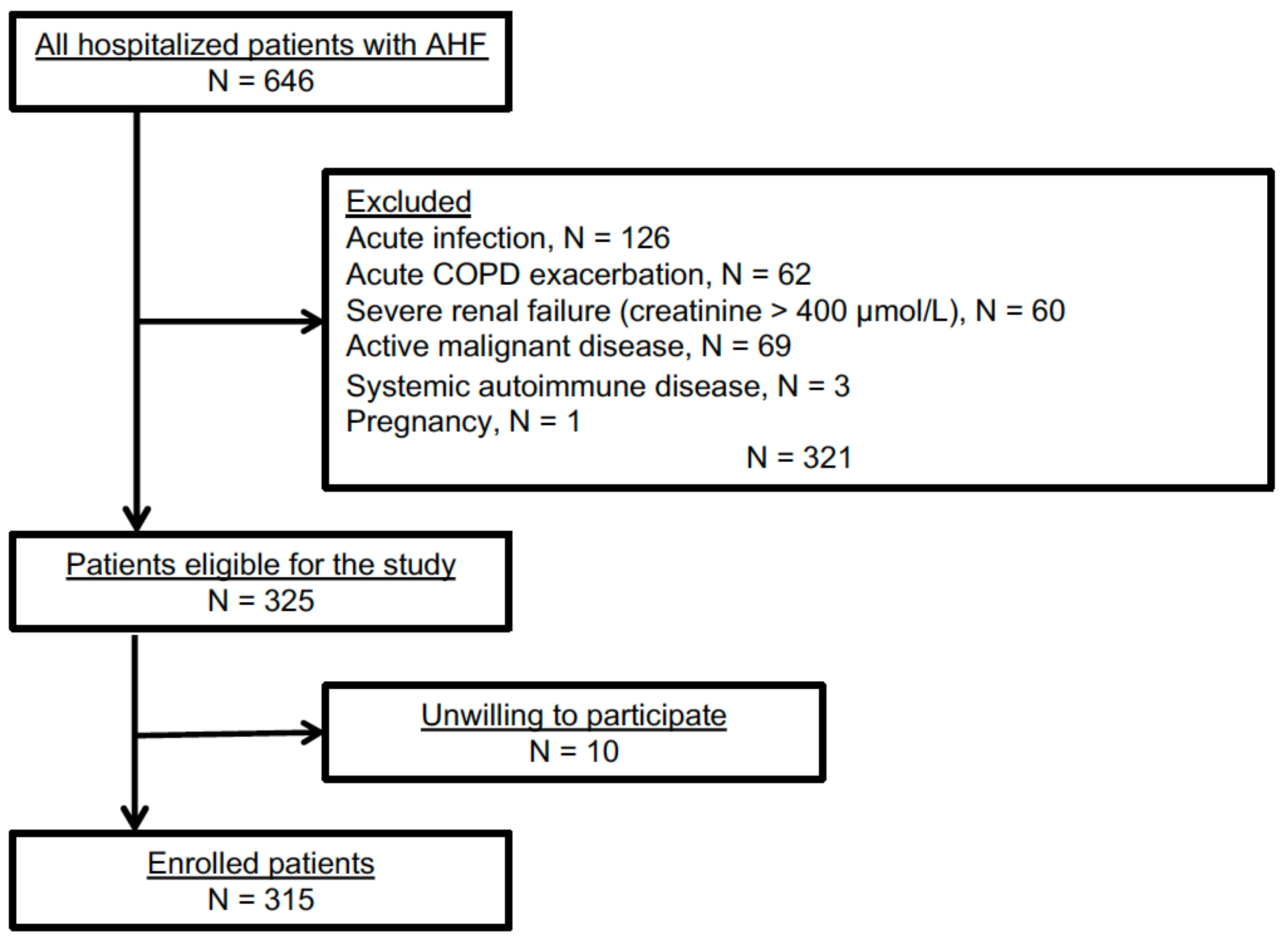
Study flowchart.

Out of 646 AHF patients admitted to the Sisters of Charity University Hospital Centre, Zagreb, Croatia, between March 2018 and February 2021, 325 were eligible for the study. Of these, 10 patients were unwilling to participate (Scheme 1).

Written informed consent was obtained from each enrolled patient in compliance with Good Clinical Practice, and the investigation conforms with the principles outlined in the Declaration of Helsinki [32]. The study was approved by the local Ethics Committee of the Sisters of Charity University Hospital Centre, Zagreb, Croatia (EP 2258/18-10) and the Medical University of Graz, Austria (EK 33-258 ex 20/21). 

Patients’ demographic characteristics and chronic diseases (hypertension, diabetes mellitus type 1 and 2 (T1DM, T2DM), coronary artery disease (CAD), cardiomyopathy (CMP), atrial fibrillation (AF), chronic kidney disease (CKD), chronic obstructive pulmonary disease (COPD), and metabolic syndrome (MetS)) were recorded. Diagnoses of hypertension, diabetes mellitus, and metabolic syndrome were based on the respective guidelines [33,34,35]. Data about the onset of AHF symptoms and their evolution were given by the patients.

Detailed physical exam (vital signs, body weight, and height) with the assessment of possible signs and symptoms of pulmonary (dyspnea, orthopnea, lung rales, or crackles) or peripheral venous congestion (peripheral edema, enlarged liver, ascites, jugular vein distension) were performed at the time of admission to the hospital. Body mass index (BMI) was calculated as (body weight in kg)/(body height in m)^2^, body surface area (BSA) as 0.20247 × (body height in m)^0.725^ × (body weight in kg)^0.425^, and mean arterial pressure (MAP) as (systolic + 2 × diastolic blood pressure)/3.

A standard comprehensive transthoracic echocardiographic exam following the current American Society of Echocardiography guidelines [36] was performed within the first 24 h of hospitalization. Left ventricular ejection fraction (LVEF) was used to categorize patients as HF with preserved (HFpEF, ≥50%), mildly reduced (HFmrEF, 41–49%), or reduced ejection fraction (HFrEF, ≤40%). 

Patients were categorized into different groups or classifications according to their history of the onset, intensity, and duration of the present clinical signs and symptoms of AHF, as well as according to the revealed triggering event for the index AHF episode, (new-onset AHF vs. AHF following CHF; The New York Heart Association (NYHA) functional class; type of AHF presentation).

Participants were followed up every three months for one year after the index hospitalization for AHF. The primary end-point was all-cause mortality after one year. The patients’ vital status was obtained via direct telephone contact, from the available clinical records, or from the registries of the Croatian Health System.

### 2.2. Laboratory Procedures

Venous blood was obtained from the AHF patients on admission to the hospital. The fasting status of patients at admission was unknown. The blood was collected in 6 mL tubes with a VACUETTE^®^ Z Serum Clot Activator (Greiner Bio-one GmbH, Kremsmuenster, Austria). The tubes were incubated for 30 min at room temperature and subsequently centrifuged at 1800× *g* for 10 min at 4 °C. Routine laboratory analyses including serum glucose, total protein, albumin, bilirubin, creatinine, urea, sodium, potassium, chloride, alanine aminotransferase (ALT), aspartate aminotransferase (AST), lactate dehydrogenase (LDH), and creatine kinase (CK) were measured using ARCHITECT c8000, Abbott 2013 (Chicago, IL, USA). High-sensitivity troponin I (hsTnI) was measured using ARCHITECT i2000SR, Abbott 2013 (Chicago, IL, USA). C-reactive protein (CRP), total cholesterol, high-density lipoprotein cholesterol (HDL-C), and triglycerides were measured by using the Cobas c system (Roche Diagnostics, Hitachi, Tokyo, Japan) and low-density lipoprotein cholesterol (LDL-C) was calculated using Friedewald’s formula [37]. Interleukin-6 (IL-6) and N-terminal pro-brain natriuretic peptide (NT-proBNP) were quantified via electrochemiluminescence immunoassay using the Cobas e801 system (Roche Diagnostics, Hitachi, Tokyo, Japan). Glomerular filtration rate (eGFR) was calculated according to Levey et al. [38].

Blood cell counts and hemoglobin were measured in full blood supplemented with K3EDTA with an Automated Hematology Analyzer (XN-1000), Sysmex Corporation (Kobe, Japan), and UniCel DxH 900 Coulter Cellular Analysis System, Beckman Coulter (Brea, CA, USA).

Blood collected into VACUETTE tubes containing 3.2% sodium citrate was used for fibrinogen quantification as well as for determination of international normalized ratio (INR), which was calculated based on prothrombin time determined with the BCS XP System, Siemens, (Marburg, Germany).

For acid–base status, arterial blood was collected with RapidLyte syringes containing electrolyte-balanced heparin (23 IU). The partial pressure of carbon dioxide (pCO_2_), bicarbonate (HCO_3_^−^), partial pressure of oxygen (pO_2_), and oxygen saturation (sO_2_) were measured with the RAPID Point Blood Gas System, Siemens 2013, and an ABL800 FLEX Blood gas Analyzer, Radiometer 2020.

### 2.3. Lipoprotein Profiling via Nuclear Magnetic Resonance (NMR) Spectroscopy 

Serum levels of total HDL-apoA-II and HDL-apoA-I, as well as of their size/density subfractions (HDL1: 1.063–1.100 kg/L; HDL2: 1.100–1.112 kg/L; HDL3: 1.112–1.125 kg/L; HDL4: 1.125–1.210 kg/L), were determined on a Bruker 600 MHz Avance Neo NMR spectrometer using the Bruker IVDr lipoprotein subclass analysis protocol, as described in [39]. The *AXINON^®^ lipoFIT^®^-*S100 test system (Numares Health, Regensburg, Germany) was used for the measurements of serum levels of the total, large, and small HDL-p (HDL-p, LHDL-p, and SHDL-p), as previously described [31].

### 2.4. Statistics

Metric parameters are summarized as mean and standard deviation (SD) or median and minimum to maximum, whereas absolute and relative frequencies were used to describe categorical parameters. Differences in patients who survived and those who died within 1 year as well as between groups defined by various clinical characteristics were tested with a *t*-test, Mann–Whitney U test, or Fisher’s exact test. Univariable and multivariable Cox regression analyses were used to examine the association between HDL parameters and 1-year mortality. In the multivariable analyses, we adjusted for age, sex, BMI, MAP, eGFR, blood urea nitrogen (BUN), CRP, NT-proBNP, hemoglobin, ALT, albumin, and total cholesterol. Results are presented as hazard ratio (HR) and the respective 95% confidence interval (CI) per 1 SD increase. We performed Kaplan–Meier survival analysis for the tertiles of the HDL parameters significantly associated with mortality in the multivariable analysis and compared them with the log-rank test. Receiver operating characteristic curve analysis was performed to assess the prognostic ability of the HDL parameters. The Spearman correlation coefficient was used to assess correlations between HDL parameters and various clinical and laboratory parameters. Results are presented in a heatmap. A *p*-value < 0.05 was considered significant. R version 4.1.0 was used for these analyses.

## 3. Results

### 3.1. Clinical Characteristics and Medication 

This study comprised 315 hospitalized AHF patients with a mean (±standard deviation) patient age of 74.2 ± 10.5 years, 136 (43.2%) of whom were female. Arterial hypertension and previously known CMP were the most frequent comorbidities (93.3% and 91.4%, respectively), followed by MetS (68.9%), AF (54.0%), chronic CAD (49.5%), CKD (45.4%), and T2DM (41.9%). At the time of admission to the hospital, 298 (94.6%) presented as NYHA 4 functional class, with a median (range) symptom duration of 5 (1–5) days before hospitalization. Almost half of the patients (47.2%) had reduced left ventricular ejection fraction. In total, 118 patients (37.5%) died within 1 year after the index episode of AHF. Compared with those who were alive at the 1-year follow-up, deceased patients were significantly older, more frequently suffered from chronic CMP and CKD, and had higher chronic use of furosemide. Long-term use of acetylsalicylic acid was more frequent among the deceased, suggesting a higher prevalence of cerebrovascular and peripheral arterial disease in that patient group. At the time of hospital admission, the deceased patients had a significantly lower MAP and heart rate, more pronounced signs of right ventricular failure, and a longer delay between symptom onset and presentation to the hospital. There was no significant difference in LVEF between those who survived or died within 1 year after the index episode of AHF, but the deceased patients had a significantly higher systolic pulmonary artery pressure (SPAP) and significantly increased interventricular septum thickness and left ventricular posterior wall thickness at the time of admission to the hospital (Table 1 and Table S1).

### 3.2. Laboratory Parameters

Baseline serum levels of albumin, total cholesterol, LDL-C, HDL-C, sodium, chloride, erythrocytes, ALT, and hemoglobin, as well as eGFR, were significantly lower, whereas BUN, creatinine, NT-proBNP, and CRP were significantly higher in patients who died than in those who were alive within 1 year after hospitalization for AHF (Table 2). There was no significant difference between the groups regarding AST, CK, LDH, potassium, hsTnI, IL-6, triglycerides, and INR, as well as glucose, protein, bilirubin, leukocytes, platelets, fibrinogen, pH, pO_2_, sO_2_, pCO_2_, and HCO_3_^−^ (Table 2 and Table S2).

### 3.3. Association between HDL Parameters and 1-Year Mortality in AHF Patients

Serum levels of total HDL-apoA-II and HDL-apoA-I, and subfractions thereof, as well as of HDL-p, LHDL-p, and SHDL-p are shown in Table S3. In the univariable Cox regression analyses, decreased serum levels of HDL-apoA-II (and its subfractions HDL2-apoA-II, HDL3-apoA-II, and HDL4-apoA-II) and HDL-apoA-I (and its subfractions HDL2-apoA-I, HDL3-apoA-I, and HDL4-apoA-I), as well as of HDL-p and SHDL-p were significantly associated with increased 1-year mortality in AHF patients (Table 3). However, only the association between HDL-apoA-II, HDL2-apoA-II, and HDL3-apoA-II remained significant after adjustment for age, sex, and the clinical and laboratory parameters significantly associated with 1-year mortality in the univariable analyses (Table 3 and Table S4).

**Figure 1:**
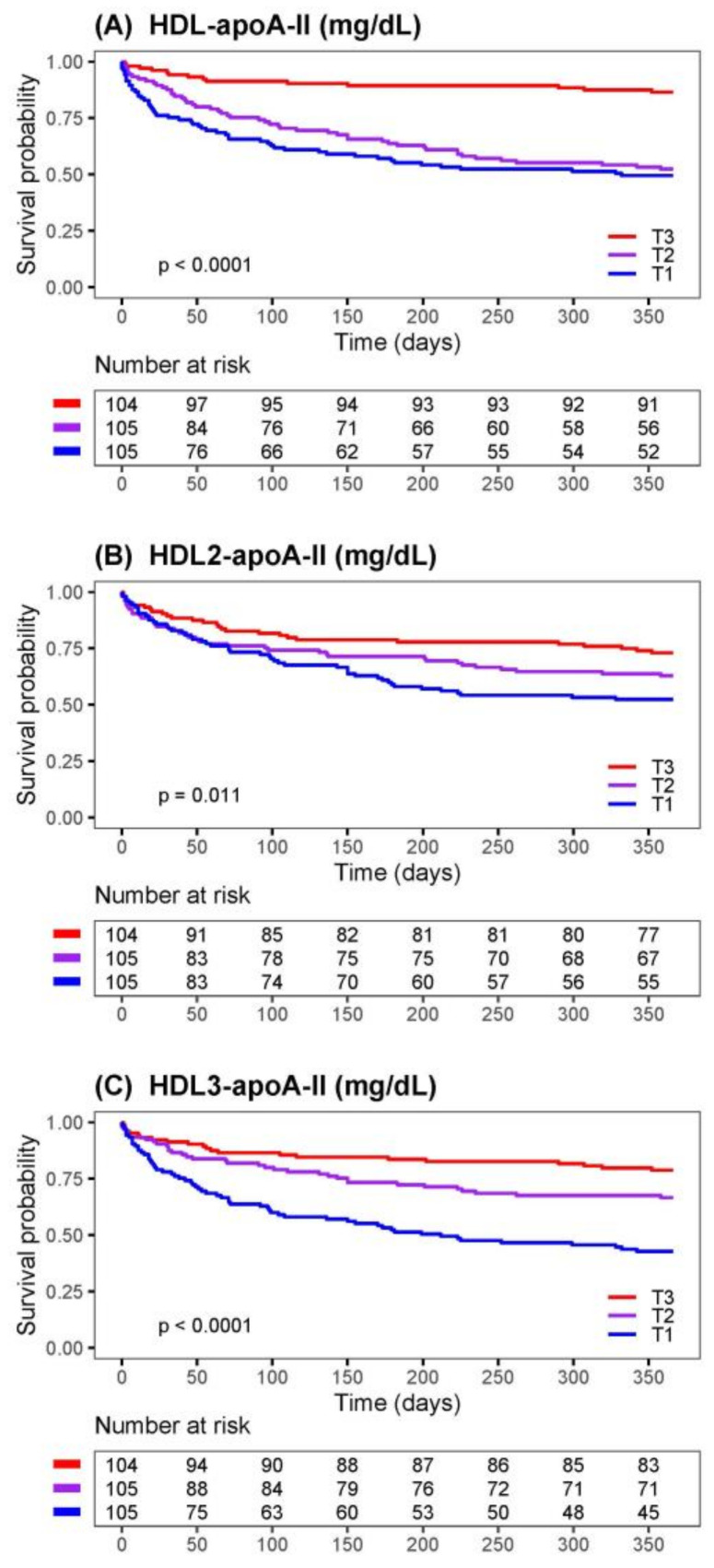
Risk of 1-year mortality of AHF patients stratified by tertiles of HDL parameters. Kaplan–Meier survival curves for 1-year mortality of AHF patients comparing tertiles of (**A**) HDL-apoA-II, (**B**) HDL2-apo-AII, and (**C**) HDL3-apoA-II. The number of patients at risk at each time point is presented below the graphs. The limits of the tertiles (T) in mg/dL: HDL-apoA-II (T1 = 7.84–21.67, T2 = 21.67–27.02, T3 = 27.02–49.23); HDL2-apoAII (T1 = 1.30–2.81, T2 = 2.81–3.55, T3 = 3.55–7.70); HDL3-apoA-II (T1 = 1.15–4.17, T2 = 4.17–5.55, T3 = 5.55–13.55).

Kaplan–Meier survival plots comparing tertiles of HDL-apoA-II, HDL2-apoA-II, and HDL3-apoA-II are shown in Figure 1. The differences between the tertiles were significant for the three tested HDL parameters; the patients within the highest tertile (T3) had the best survival, and those in the lowest tertile (T1) had the worst.

**Figure 2:**
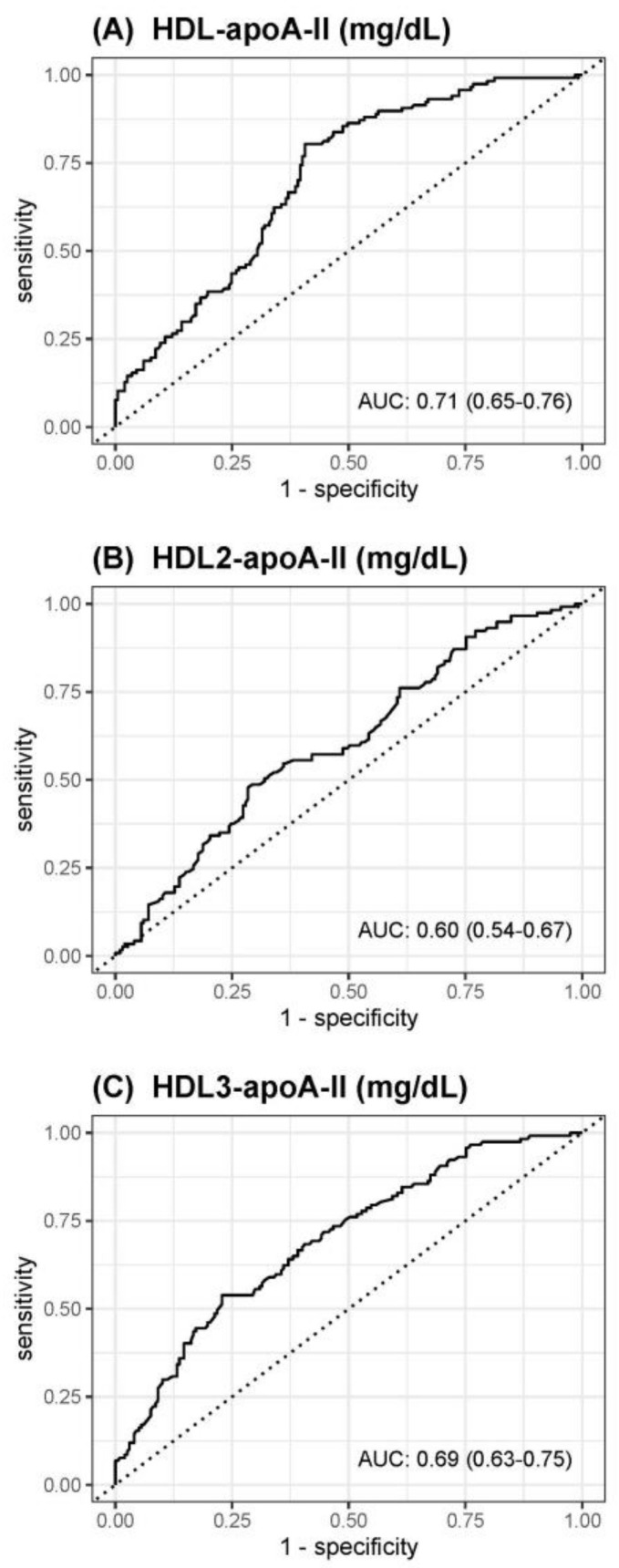
Receiver operating characteristics curves for prediction of death within 1 year of (**A**) HDL-apoA-II, (**B**) HDL2-apoA-II, or (**C**) HDL3-apoA-II.

The receiver operating characteristics curve analyses to assess the accuracy of HDL-apoA-II, HDL2-apoA-II, or HDL3-apoA-II to predict death within 1 year are shown in Figure 2. The best predictive value with an area under the curve (AUC) of 0.71 was observed for HDL-apoA-II, followed by HDL3-apoA-II (AUC 0.69) and HDL2-apoA-II (AUC 0.60).

### 3.4. Correlation Analyses of HDL-apoA-II, HDL2-apoA-II, and HDL3-apoA-II with Clinical and Laboratory Parameters

**Figure 3:**
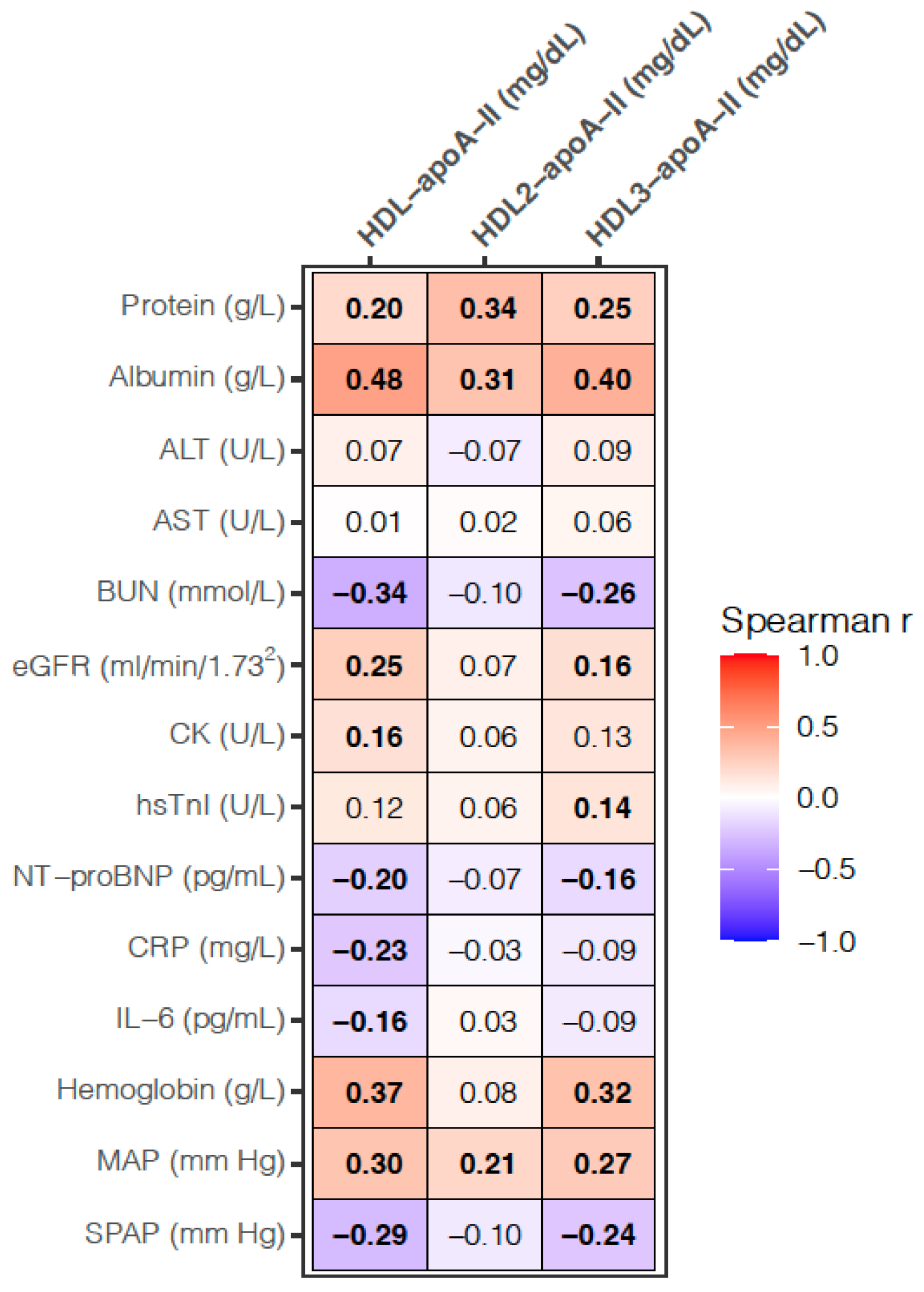
Heatmap for correlation analyses between the HDL and laboratory, as well as clinical parameters. Values presented are the Spearman correlation coefficient. *p*-values < 0.017 are considered significant after a Bonferroni correction for multiple testing and significant correlations are depicted in bold. SPAP was measured in 259 patients; otherwise, the analyses are based on 314 samples. BUN, blood urea nitrogen; CK, creatine kinase; CRP, C-reactive protein; eGFR, estimated glomerular filtration rate; hsTnI, high-sensitivity troponin I; MAP, mean arterial pressure; NT-proBNP, N-terminal pro-brain natriuretic peptide; SPAP, systolic pulmonary artery pressure.

HDL-apoA-II, HDL2-apoA-II, and HDL3-apoA-II were significantly positively correlated with serum proteins, albumin, and MAP. Both HDL-apoA-II and HDL3-apoA-II were significantly positively correlated with eGFR and hemoglobin and negatively with BUN, NT-proBNP, and SPAP. In comparison to HDL-apoA-II, which was significantly positively correlated with CK and negatively with CRP and IL-6, HDL3-apoA-II was significantly positively correlated with hsTnI. Neither of the tested parameters was correlated with ALT or AST (Figure 3).

### 3.5. Differences in HDL-apoA-II, HDL2-apoA-II, and HDL3-apoA-II in Various Groups of AHF Patients

Serum levels of HDL-apoA-II, HDL2-apoA-II, and HDL3-apoA-II were significantly lower in patients with sign(s) of venous volume overload (enlarged liver, peripheral edema, ascites, or jugular venous distension) than in those without, as well as in patients who developed AHF on the top of CHF compared with those developing new-onset AHF (Table 4). While both HDL-apoA-II and HDL3-apoA-II were significantly higher in patients with CAD and significantly lower in patients with AF, the levels of HDL-apoA-II were also significantly lower in patients with MetS than those without these comorbidities (Table 4).

## 4. Discussion

Despite established multivariable predictive models comprising patients’ characteristics, clinical signs, and serum biomarkers, the estimation of risk in AHF is difficult, and it is associated with poor patient outcomes [40,41]. Therefore, the identification of new biomarkers, reflecting different aspects of the complex underlying AHF pathophysiology, may improve risk assessment and thus help physicians in initiating appropriate therapeutic interventions and improve the overall management of AHF patients [42]. Previously, we and others identified the prognostic capacity of HDL-p and SHDL-p in AHF and CHF patients [28,31].

In the present study, we showed a strong association between HDL-apoA-II and its small/dense subfractions HDL2-apoA-II and HDL3-apoA-II with 1-year mortality in AHF patients (Table 3). These associations were not significantly affected by medication (Table S5). A significant negative association between the HDL-apoA-II/HDL-apoA-I ratio and 1-year mortality in uni- and multivariable Cox regression (univariable HR 0.72 per 1 SD increase, 95% CI 0.59–0.87, *p* < 0.001; multivariable HR 0.80 per 1 SD increase, 95% CI 0.64–1.00, *p* = 0.046, adjusted for the variables used in Table 3), suggests that this parameter might also be of prognostic value in AHF. The associations of HDL-p, SHDL-p, and HDL-apoA-I with 1-year mortality were weaker and only significant in a multivariable Cox regression model, which was adjusted for fewer covariables than presented here, similar to our previous study in which we evaluated the prognostic capacity of HDL-p [31] (Table S6). The significant inverse association between HDL-apoA-I and 1-year mortality observed in the present study (Table S6) is consistent with a previously observed association between low serum- and HDL-apoA-I levels and increased mortality in CHF and AHF patients [29,30,43]. Notably, in these studies, the multivariable models were similar to our minimally adjusted model presented in Table S6. In such a minimally adjusted model, HDL-C was neither associated with 1-year mortality in the present (HR 1.05 per 1 SD increase, 95% CI 0.82–1.33, *p* = 0.718) nor with 3-month mortality in our previous study [31].

A recent study reported a positive association between HDL particle size, as well as HDL cholesterol content (calculated as HDL-C/HDL-p ratio), and cardiovascular death in CHF patients [44]. In the present study, univariable Cox regression analysis revealed a significant positive association of the HDL-C/HDL-apoA-II ratio (HR 1.37 per 1 SD increase, 95% CI 1.15–1.62, *p* < 0.001), but not the HDL-C/apoA-I ratio (HR 1.16 per 1 SD increase, 95% CI 0.96–1.41, *p* = 0.134), with 1-year mortality. The association between the HDL-C/HDL-apoA-II ratio and 1-year mortality remained significant in our fully adjusted model (HR 1.30 per 1 SD increase, 95% CI 1.07–1.59, *p* = 0.008), suggesting a prognostic value of the cholesterol load of HDL particles containing apoA-II (but not HDL particles containing only apoA-I) in AHF patients.

HDL-apoA-II, as determined via NMR spectroscopy, represents two subpopulations of HDL particles—namely, a subpopulation containing both apoA-I and apoA-II, as well as a minor HDL subpopulation containing only apoA-II. HDL-apoA-I represents the HDL subpopulation containing both apoA-I and apoA-II, as well as a subpopulation containing only apoA-I [10,12,14]. Accordingly, the prognostic value of HDL-apoA-I reflects the composite contribution of the whole spectrum of HDL particles, with exception of a minor subpopulation containing only apoA-II. Considering this, the weaker prognostic value observed in the present study in terms of HDL-apoA-I, compared with the prognostic value of HDL-apoA-II, might reflect the lack of contribution of a minor subpopulation of HDL containing only apoA-II, or the weakening effect of the subset of HDL particles containing only apoA-I. Since the determination of the serum levels of the subset of HDL particles containing only apoA-II or only apoA-I requires physical separation of the particles, which is beyond the scope of the study, the prognostic value of these subsets of HDL particles could not be evaluated.

We observed a positive correlation between HDL-apoA-II and the biomarkers for the biosynthetic activity of the liver and renal function, both of which are impaired in HF due to hypoperfusion and/or congestion [4,45]. Furthermore, we observed a positive correlation with CK, whose decreased serum levels reflect catabolic dominance and muscle wasting, the typical signs of advanced HF [46], as well as a negative correlation with NT-proBNP, an established marker of heart failure and left ventricular dysfunction [47]. This indicates a profound sensitivity of HDL-apoA-II to the AHF pathophysiology, with a markedly more profound sensitivity of HDL3-apoA-II, compared with HDL2-apoA-II. Consistently, serum levels of HDL-apoA-II and HDL3-apoA-II were lower in more severe AHF cases, such as those with venous congestion, MetS, or AF, than those without, as well as in the patients who developed AHF on top of CHF, compared with the new-onset AHF cases [48]. Despite the profound association of HDL-apoA-I, HDL-p, and SHDL-p with markers of AHF severity (Table S7), HDL-apoA-II and its subfractions 2 and 3 seem to be superior sensors of the underlying AHF pathophysiology, manifested in a strong association between these parameters and 1-year mortality in AHF patients.

It is well-established that functional, healthy HDL exerts numerous protective effects on the cardiovascular system. These include cholesterol efflux from the blood vessel wall macrophages, antioxidant and anti-inflammatory activities in blood vessels and myocardium, nitric oxide (NO) induction, and the maintenance of a normal endothelial function in systemic and cardiac circulation (reviewed in [49]). Furthermore, HDL attenuates cardiomyocyte hypertrophy and myocardial fibrosis and improves signs and symptoms of HF in mouse models of HF [25,27,50]. Contrary to cell culture experiments, which provide unequivocal evidence of the diminishing effect of apoA-II on the capacity of HDL to induce endothelial NO production [20,51], studies in transgenic models generated contradictory results regarding the impact of apoA-II on HDL function. While overexpression of human apoA-II in mice increased atherosclerosis susceptibility [52] and converted HDL to proinflammatory particles with a diminished antioxidant capacity [23,24], overexpression of human apoA-II in rabbits reduced aortic and coronary atherosclerosis, accompanied by an increased cholesterol efflux and antioxidant activities of HDL [53]. In the present study, significantly higher serum levels of HDL-apoA-II and HDL3-apoA-II (as well as SHDL-p) (Table 4 and Table S8) were observed in AHF patients with CAD than in those without, suggesting a pro-atherogenic activity of these subsets of HDL particles. Previous studies, however, found an inverse association between plasma levels of HDL containing both apoA-I and apoA-II and coronary heart disease [54,55]. The positive association between HDL-apo-AII (as well as SHDL-p) and CAD in the present study might be due to a dysregulation of HDL metabolism by the complex underlying AHF pathophysiology. The observed negative correlation between HDL-apoA-II (but not of its subfractions HDL2-apoA-II and HDL3-apoA-II) and inflammatory markers (CRP and IL-6) in the present study might reflect the diminishing effect of inflammation-driven reduction in HDL-apoA-II availability, thus contrasting the previously established positive association between plasma levels of HDL particles containing apoA-II and inflammatory response in patients with ST-elevation myocardial infarction [21]. Despite the already accumulated knowledge on the functionality of HDL containing apoA-II, intervention experiments in the rodent models of HF, such as treatment of animals with apoA-II enriched reconstituted HDL or apoA-II overexpression, are required to gain mechanistic insights into whether and how HDL-apoA-II affects HF pathophysiology, the function of the failing heart, and HF mortality.

The major strengths of the study include well-defined patients, uniformly collected, stored, and analyzed serum samples, as well as comprehensive HDL profiling by using two complementary NMR spectroscopy methods. There are, however, several limitations to the present study: Due to its design, we could not examine causality for the relationship between HDL-apoA-II (and other tested HDL parameters) and clinical and laboratory parameters. Accordingly, the mechanistic relationship between HDL-apoA-II and the underlying pathophysiological processes could not be addressed. Furthermore, we could not determine the prognostic value of HDL particles containing only apoA-II, only apoA-I, or both apoA-I and apoA-II, since this requires physical separation of the particles and will, therefore, be examined in the future. Additionally, since we determined HDL parameters only on hospital admission, the impact of therapeutic intervention as well as any possible temporal development could not be examined. Considering the rather moderate number of available samples (*n* = 315), our results still need to be confirmed in larger AHF cohorts.

## 5. Conclusions

Based on our results, we conclude that low baseline serum levels of HDL-apoA-II, HDL2-apoA-II, and HDL3-apoA-II are associated with increased 1-year mortality in AHF patients and might, therefore, be of prognostic value in AHF.

## Figures and Tables

**Table 1:** Baseline characteristics of AHF patients.

	Alive (*n* = 197)	Deceased (*n* = 118)	All (*n* = 315)	*p*-Value
**Demographics**				
Age (years)	72.5 (10.4)	77.0 (10.1)	74.2 (10.5)	**<0.001**
Sex, Female	85 (43.1%)	51 (43.2%)	136 (43.2%)	1.000
**Comorbidities**				
Hypertension	186 (94.4%)	108 (91.5%)	294 (93.3%)	0.355
T1DM	2 (1.0%)	2 (1.7%)	4 (1.3%)	0.632
T2DM	76 (38.6%)	56 (47.5%)	132 (41.9%)	0.127
CAD	100 (50.8%)	56 (47.5%)	156 (49.5%)	0.642
CMP	173 (87.8%)	115 (97.5%)	288 (91.4%)	**0.003**
AF	98 (49.7%)	72 (61.0%)	170 (54.0%)	0.062
CKD	72 (36.5%)	71 (60.2%)	143 (45.4%)	**<0.001**
COPD	45 (22.8%)	39 (33.1%)	84 (26.7%)	0.050
MetS	130 (66.0%)	87 (73.7%)	217 (68.9%)	0.168
**AHF type**				**0.003**
New onset AHF	24 (12.2%)	3 (2.5)%	27 (8.6%)	
AHF following CHF	173 (87.8%)	115 (97.5%)	288 (91.4%)	
**Physical examination at admission**				
BMI (kg/m^2^)	27.4 (17.0–46.7)	29.1 (18.4–65.3)	28.0 (17.0–65.3)	0.067
MAP (mmHg)	108.1 (24.2)	96.0 (19.5)	103.6 (23.3)	**<0.001**
Heart rate (beats/min)	103.8 (25.8)	95.3 (27.5)	100.6 (26.7)	**0.006**
Respiratory rate (breaths/min)	29.3 (6.9)	28.8 (6.0)	29.1 (6.5)	0.474
**Signs and symptoms**				
Symptom duration (days)	5.0 (1.0–5.0)	5.0 (1.0–5.0)	5.0 (1.0–5.0)	**0.022**
Rales or crackles	193 (98.0%)	118 (100.0%)	311 (98.7%)	0.301
Dyspnoea	196 (99.5%)	118 (100%)	314 (99.7%)	1.000
Orthopnoea	184 (93.4%)	115 (97.5%)	299 (94.9%)	0.183
JVD	97 (49.2%)	77 (65.3%)	174 (55.2%)	**0.007**
Enlarged liver	95 (48.2%)	81 (68.6%)	176 (55.9%)	**<0.001**
Peripheral edema	114 (57.9%)	90 (76.3%)	204 (64.8%)	**<0.001**
Ascites	20 (10.2%)	29 (24.6%)	49 (15.6%)	**0.001**
**NYHA class**				0.305
3	13 (6.6%)	4 (3.4%)	17 (5.4%)	
4	184 (93.4%)	114 (96.6%)	298 (94.6%)	
**AHF presentation**				**0.030**
Worsening of CHF	118 (59.9%)	90 (76.3%)	208 (66.0%)	
Hypertensive AHF	27 (13.7%)	7 (5.9%)	34 (10.8%)	
Isolated right-sided HF	3 (1.5%)	0 (0.0%)	3 (1.0%)	
ACS and AHF	30 (15.2%)	13 (11.0%)	43 (13.7%)	
Pulmonary edema	15 (7.6%)	8 (6.8%)	23 (7.3%)	
Cardiogenic shock	4 (2.0%)	0 (0.0%)	4 (1.3%)	
**AHF class**				0.575
HFrEF, EF < 40%	88 (44.9%)	55 (51.4%)	143 (47.2%)	
HFmrEF, EF 41–49%	55 (28.1%)	26 (24.3%)	81 (26.7%)	
HFpEF, EF ≥ 50%	53 (27.3)	26 (24.3%)	79 (26.1%)	
**Echocardiography**				
LVEDd/BSA (mm/m^2^)	29.1 (4.9)	28.5 (5.2)	28.8 (5.0)	0.346
IVS (mm)	12.7 (2.1)	13.3 (2.4)	12.9 (2.2)	**0.038**
PW (mm)	12.5 (1.9)	13.1 (1.8)	12.7 (1.9)	**0.016**
LVEF (%)	40.1 (11.9)	39.1 (12.6)	39.8 (12.1)	0.455
SPAP (mmHg)	47.0 (30.0–90.0)	50.0 (30.0–102.0)	50.0 (30.0–102.0)	**0.005**

Data are presented as *n* (%), mean and standard deviation, or as median and minimum to maximum. Differences between AHF patients who survived and those who died within 1 year after study inclusion were tested with the t or Mann–Whitney U test or Fisher’s exact test. *p*-values < 0.05 are considered significant and are depicted in bold. ACS, acute coronary syndrome; AF, atrial fibrillation; AHF, acute heart failure; BMI, body mass index; CAD, coronary artery disease; CHF, chronic heart failure; CKD, chronic kidney disease; CMP, cardiomyopathy; COPD, chronic obstructive pulmonary disease; HFrEF, heart failure with reduced ejection fraction; HFmrEF, heart failure with mildly reduced ejection fraction; HFpEF, heart failure with preserved ejection fraction; IVS, interventricular septum thickness; JVD, jugular vein distension; LVEDd, left ventricular end-diastolic diameter; LVEF, left ventricular ejection fraction; PW, left ventricular posterior wall thickness; MAP, mean arterial pressure; MetS, metabolic syndrome; NYHA, New York Heart Association Functional Classification; SPAP, systolic pulmonary artery pressure; T1DM, diabetes mellitus Type 1; T2DM, diabetes mellitus Type 2.

**Table 2:** Laboratory data of AHF patients upon hospital admission.

	Alive (*n* = 197)	Deceased (*n* = 118)	All (*n* = 315)	*p*-Value
Albumin (g/L)	38.2 (19.7–48.9)	36.7 (21.8–56.5)	37.8 (19.7–56.5)	**0.017**
AST (U/L)	28.0 (10.0–1720.0)	27.0 (6.0–1080.0)	28.0 (6.0–1720.0)	0.427
ALT (U/L)	25.0 (7.0–1082.0)	21.0 (6.0–1168.0)	25.0 (6.0–1168.0)	**0.031**
CK (U/L)	105.0 (13.0–8135.0)	78.0 (20.0–2414.0)	93.0 (13.0–8135.0)	0.198
LDH (U/L)	252.0 (119.0–3401.0)	283.0 (111.0–2753.0)	265.0 (111.0–3401.0)	0.148
BUN (mmol/L)	8.3 (3.0–34.7)	12.3 (3.1–32.2)	9.6 (3.0–34.7)	**<0.001**
Creatinine (µmol/L)	107.0 (58.0–303.0)	131.5 (59.0–366.0)	117.0 (58.0–366.0)	**<0.001**
eGFR (mL/min/1.73 m^2^)	54.0 (12.1–103.5)	38.4 (10.8–98.5)	46.6 (10.8–103.5)	**<0.001**
Sodium (mmol/L)	140.0 (112.0–147.0)	138.0 (122.0–148.0)	140.0 (112.0–148.0)	**<0.001**
Potassium (mmol/L)	4.5 (3.2–6.3)	4.5 (3.1–7.1)	4.5 (3.1–7.1)	0.091
Chloride (mmol/L)	104.0 (78.0–115.0)	100.0 (82.0–112.0)	103.0 (78.0–115.0)	**<0.001**
hsTnI (ng/L)	39.0 (10.0–50,000.0)	61.0 (10.0–32,364.0)	46.0 (10.0–50,000.0)	0.898
NT-proBNP (pg/mL)	5350.0 (263.0–70,000.0)	10,733.0 (226.0–70,000.0)	6692.0 (226.0–70,000.0)	**<0.001**
CRP (mg/L)	10.3 (0.6–246.5)	24.9 (0.7–240.0)	12.2 (0.6–246.5)	**<0.001**
IL-6 (pg/mL)	22.1 (2.4–1848.0)	40.6 (3.4–2800.0)	25.1 (2.4–2800.0)	0.136
TC (mmol/L)	3.8 (1.8–9.6)	3.3 (1.6–8.0)	3.5 (1.6–9.6)	**<0.001**
HDL-C (mmol/L)	1.1 (0.4–3.0)	1.1 (0.2–2.5)	1.1 (0.2–3.0)	**0.025**
LDL-C (mmol/L)	2.0 (0.5–7.9)	1.7 (0.6–5.9)	1.9 (0.5–7.9)	**<0.001**
Triglycerides (mmol/L)	1.3 (0.6–8.9)	1.3 (0.6–2.1)	1.3 (0.6–8.9)	0.064
Erythrocytes (×10^12^/L)	4.7 (3.3–6.3)	4.4 (2.7–6.4)	4.6 (2.7–6.4)	**<0.001**
Hemoglobin (g/L)	138.0 (93.0–185.0)	126.0 (54.0–170.0)	134.0 (54.0–185.0)	**<0.001**
INR	1.2 (0.8–5.3)	1.3 (0.8–6.1)	1.2 (0.8–6.1)	0.077

Data are presented as median and minimum to maximum. Differences between AHF patients who were alive and those who died within 1 year after study inclusion were tested with the Mann–Whitney U test. *p*-values < 0.05 are considered significant and are depicted in bold. ALT, alanine aminotransferase; AST, aspartate aminotransferase; BUN, blood urea nitrogen; CK, creatine kinase; CRP, C-reactive protein; eGFR, estimated glomerular filtration rate; hsTnI, high-sensitivity troponin I; HDL-C, high-density lipoprotein cholesterol; IL-6, interleukin-6; INR, international normalized ratio; LDH, lactate dehydrogenase; LDL-C, low-density lipoprotein cholesterol; NT-proBNP, N-terminal pro-brain natriuretic peptide; TC, total cholesterol.

**Table 3:** Cox regression analyses of HDL parameters as predictors of 1-year mortality in AHF patients.

		Univariable			Adjusted *		
	SD	HR (95% CI) per 1 SD	*p*-Value	Events/*n*	HR (95% CI) per 1 SD	*p*-Value	Events/*n*
**HDL-apoA-II**	6.2	0.50 (0.41–0.62)	**<0.001**	117/314	0.67 (0.47–0.94)	**0.020**	111/302
HDL1-apoA-II	1.5	1.05 (0.89–1.25)	0.559	117/314	1.06 (0.85–1.32)	0.600	111/302
**HDL2-apoA-II**	0.9	0.73 (0.60–0.89)	**0.002**	117/314	0.72 (0.54–0.95)	**0.019**	111/302
**HDL3-apoA-II**	1.6	0.51 (0.40–0.63)	**<0.001**	117/314	0.59 (0.43–0.80)	**<0.001**	111/302
HDL4-apoA-II	5.0	0.51 (0.42–0.63)	**<0.001**	117/314	0.81 (0.60–1.10)	0.185	111/302
HDL-apoA-I	29.4	0.66 (0.54–0.80)	**<0.001**	117/314	1.00 (0.76–1.33)	0.980	111/302
HDL1-apoA-I	16.4	1.12 (0.95–1.32)	0.172	117/314	1.13 (0.92–1.39)	0.241	111/302
HDL2-apoA-I	4.6	0.77 (0.63–0.94)	**0.011**	117/314	0.98 (0.77–1.24)	0.839	111/302
HDL3-apoA-I	6.1	0.56 (0.46–0.69)	**<0.001**	117/314	0.79 (0.60–1.04)	0.096	111/302
HDL4-apoA-I	16.5	0.53 (0.43–0.65)	**<0.001**	117/314	0.92 (0.67–1.28)	0.632	111/302
HDL-p	8.3	0.53 (0.43–0.65)	**<0.001**	118/314	0.77 (0.55–1.07)	0.114	112/302
LHDL-p	3.3	0.98 (0.81–1.19)	0.836	100/268	1.17 (0.94–1.46)	0.150	97/262
SHDL-p	7.4	0.56 (0.44–0.70)	**<0.001**	101/290	0.85 (0.62–1.18)	0.330	97/280

Parameters significantly associated with 1-year mortality after adjustment as well as significant *p*-values (<0.05) are depicted in bold. Total and subfractions of HDL-apoA-II and HDL-apoA-I are given in mg/dL and HDL-p, LHDL-p, and SHDL-p in µmol/L. * Adjusted for age, sex, BMI, MAP, eGFR, BUN, CRP, NT-proBNP, hemoglobin, ALT, albumin, and total cholesterol. ALT, alanine aminotransferase; apoA-I, apolipoprotein A-I; apoA-II, apolipoprotein A-II; BMI, body mass index; BUN, blood urea nitrogen; C, cholesterol; CRP, C-reactive protein; eGFR, estimated glomerular filtration rate; HDL-p, high-density lipoprotein particle concentration; L, large; MAP, mean arterial pressure; *n*, number of observations; NT-proBNP, N-terminal pro-brain natriuretic peptide; p, particle; S, small; SD, standard deviation.

**Table 4:** Serum levels of HDL parameters in various groups of AHF patients.

		HDL-apoA-II (mg/dL)	HDL2-apoA-II (mg/dL)	HDL3-apoA-II (mg/dL)
CAD	no (*n* = 158)	23.3 (7.8–44.6)	3.2 (1.3–6.4)	4.5 (1.2–9.8)
	yes (*n* = 156)	25.2 (11.5–49.2)	3.2 (1.5–7.7)	5.2 (2.0–13.6)
		***p* = 0.005**	*p* = 0.625	***p* = 0.002**
MetS	no (*n* = 98)	25.9 (12.6–40.8)	3.4 (1.8–6.4)	5.1 (1.2–8.8)
	yes (*n* = 216)	23.6 (7.8–49.2)	3.1 (1.3–7.7)	4.8 (1.2–13.6)
		***p* < 0.001**	*p* = 0.054	***p* = 0.450**
AF	no (*n* = (144)	26.3 (11.5–49.2)	3.3 (1.3–7.0)	5.4 (1.2–11.8)
	yes (*n* = 170)	22.3 (7.8–44.0)	3.0 (1.3–7.7)	4.4 (1.2–13.6)
		***p* < 0.001**	*p* = 0.100	***p* < 0.001**
Venous overload *	no (*n* = 66)	28.2 (17.2–49.2)	3.4 (1.6–7.7)	5.6 (2.9–13.6)
	yes (*n* = 248)	23.3 (7.8–40.8)	3.1 (1.3–6.4)	4.5 (1.2–9.5)
		***p* < 0.001**	***p* = 0.005**	***p* < 0.001**
AHF type	New onset AHF (*n* = 27)	29.7 (16.2–44.6)	3.5 (2.4–5.1)	6.2 (2.9–9.8)
	AHF following CHF (*n* = 287)	23.7 (7.8–49.2)	3.1 (1.3–7.7)	4.7 (1.2–13.6)
		***p* < 0.001**	***p* = 0.016**	***p* < 0.001**

Data are presented as median and minimum to maximum. Differences between the groups were tested with the Mann–Whitney U test. *p*-values < 0.017 are considered significant after a Bonferroni correction for multiple testing and are depicted in bold. AF, atrial fibrillation; AHF, acute heart failure; apoA-II, apolipoprotein A-II; CAD, coronary artery disease; CHF, chronic heart failure; HDL, high-density lipoprotein; HDL-p, HDL particle concentration; MetS, metabolic syndrome; S, small. * Any of the following: enlarged liver, peripheral edema, ascites, or jugular venous distension.

## Data Availability

Data are available within the article and supplemental material.

## Supplementary Materials

The following supporting information can be downloaded at: https://www.mdpi.com/article/10.3390/biomedicines10071668/s1, Table S1: Chronic medication of AHF patients prior to index AHF hospitalization, Table S2: Laboratory data of AHF patients upon hospital admission, Table S3: Serum levels of HDL parameters quantified by NMR spectroscopy, Table S4: Univariable Cox regression analyses of various clinical and laboratory parameters as predictors of 1-year mortality in AHF patients, Table S5: Impact of chronic medication on Cox regression analyses of HDL parameters as predictors of 1-year mortality in AHF patients, Table S6: Multivariable Cox regression analyses of HDL parameters as predictors of 1-year mortality in AHF patients, Table S7: Correlation analyses of HDL parameters with laboratory and clinical parameters, Table S8: Serum levels of HDL parameters in various groups of AHF patients.
