# Supplementary material for: HDL-apoA-II Is Strongly Associated with 1-Year Mortality in Acute Heart Failure Patients
Source: Biomedicines. 2022 Jul 11;10(7):1668. doi: 10.3390/biomedicines10071668 (PMC9313377; doi:10.3390/biomedicines10071668)
Supplement: Supplementary file 1 [file biomedicines-10-01668-s001.zip › Table S2.pdf]

Table S2. Laboratory data of AHF patients upon hospital admission

	Alive (N=197)	Deceased (N=118)	All (N=315)	p-value
Glucose (mmol/L)	7.7 (3.0-34.8)	8.1 (3.6-33.9)	7.9 (3.0-34.8)	0.475
Total proteins (g/L)	67.0 (41.0-87.0)	65.5 (43.0-81.0)	67.0 (41.0-87.0)	0.227
Bilirubin (μmol/L)	17.4 (3.9-84.2)	17.2 (4.3-114.8)	17.3 (3.9-114.8)	0.056
Leukocytes (x 10^9/L)	9.5 (3.2-42.5)	10.4 (3.7-23.0)	9.9 (3.2-42.5)	0.785
Platelets (x 10^9/L)	224.0 (39.0-556.0)	222.5 (48.0-798.0)	224.0 (39.0-798.0)	0.454
Fibrinogen (g/L)	4.0 (1.8-9.8)	4.0 (1.0-8.3)	4.0 (1.0-9.8)	0.244
pH	7.4 (7.0-7.5)	7.4 (6.8-7.5)	7.4 (6.8-7.5)	0.653
pO_2 (kPa)	8.8 (4.1-19.5)	8.8 (4.6-23.8)	8.8 (4.1-23.8)	0.658
sO_2 (%)	93.0 (47.0-99.4)	92.3 (60.0-99.0)	93.0 (47.0-99.4)	0.777
pCO_2 (kPa)	5.2 (2.9-14.0)	5.2 (2.6-21.9)	5.2 (2.6-21.9)	0.185
HCO_3 (mmol/L)	23.9 (9.8-42.2)	24.4 (11.3-47.9)	23.9 (9.8-47.9)	0.259

Data are presented as median and range (minimum to maximum). Differences between AHF patients who were alive and those who died within 1 year after study inclusion were tested with the Mann-Whitney U test.

P-values < 0.05 are considered significant.

pO_2, partial oxygen pressure; sO_2, oxygen saturation; pCO2, partial carbon dioxide pressure.
