# Supplementary material for: HDL-apoA-II Is Strongly Associated with 1-Year Mortality in Acute Heart Failure Patients
Source: Biomedicines. 2022 Jul 11;10(7):1668. doi: 10.3390/biomedicines10071668 (PMC9313377; doi:10.3390/biomedicines10071668)
Supplement: Supplementary file 1 [file biomedicines-10-01668-s001.zip › Table S3.pdf]

Table S3. Serum levels of HDL parameters quantified by NMR spectroscopy

HDL parameters (mg/dL)	Alive (N=197)	Deceased (N=118)	All (N=315)	p-value
HDL-apoA-II	26.4 (13.7-49.2)	22.3 (7.8-40.8)	24.3 (7.8-49.2)	**< 0.001**
HDL1-apoA-II	2.7 (0.6-10.3)	3.1 (0.3-10.3)	2.8 (0.3-10.3)	0.256
HDL2-apoA-II	3.2 (1.3-7.7)	2.9 (1.3-6.3)	3.2 (1.3-7.7)	**0.002**
HDL3-apoA-II	5.3 (2.1-13.6)	4.1 (1.2-8.8)	4.9 (1.2-13.6)	**< 0.001**
HDL4-apoA-II	12.1 (1.2-27.3)	8.5 (0.0-23.6)	10.7 (0.0-27.3)	**< 0.001**
HDL-apoA-I	128.2 (52.4-240.4)	117.2 (48.0-213.1)	123.7 (48.0-240.4)	**< 0.001**
HDL1-apoA-I	30.3 (0.5-108.7)	34.6 (2.8-101.6)	31.2 (0.5-108.7)	0.055
HDL2-apoA-I	17.2 (8.2-35.2)	16.2 (7.7-35.0)	17.1 (7.7-35.2)	**0.036**
HDL3-apoA-I	22.9 (9.9-46.1)	19.8 (2.9-35.9)	21.9 (2.9-46.1)	**< 0.001**
HDL4-apoA-I	53.2 (16.1-94.4)	43.8 (0.0-87.2)	48.3 (0.0-94.4)	**< 0.001**
HDL-p (μmol/L)	25.5 (7.2-56.3)	20.3 (4.5-41.6)	23.2 (4.5-56.3)	**< 0.001**
LHDL-p (μmol/L)	6.1 (2.8-20.5)	6.3 (2.8-17.8)	6.2 (2.8-20.5)	0.553
SHDL-p (μmol/L)	19.0 (6.1-40.4)	13.1 (6.4-35.8)	16.9 (6.1-40.4)	**< 0.001**
HDL-C/HDL-apoA-I	0.39 (0.18-0.49)	0.41 (0.25-0.49)	0.40 (0.18-0.49)	0.042
HDL-C/HDL-apoA-II	1.89 (0.52-3.46)	2.12 (0.96-4.14)	1.97 (0.52-4.14)	**< 0.001**
HDL-apoA-II/HDL-apoA-I	0.20 (0.13-0.36)	0.19 (0.10-0.30)	0.20 (0.10-0.36)	**< 0.001**

Data are presented as median and minimum to maximum. Differences between AHF patients who were alive and those who deceased within 1 year after hospitalization for AHF were tested with the Mann-Whitney U test.

P-values of < 0.05 are considered significant and are depicted in bold.

apoA-I, apolipoprotein A-I; apoA-II, apolipoprotein A-II; C, cholesterol; HDL-p, high-density lipoprotein particle concentration; L, large; p, particle; S, small.
