# Supplementary material for: HDL-apoA-II Is Strongly Associated with 1-Year Mortality in Acute Heart Failure Patients
Source: Biomedicines. 2022 Jul 11;10(7):1668. doi: 10.3390/biomedicines10071668 (PMC9313377; doi:10.3390/biomedicines10071668)
Supplement: Supplementary file 1 [file biomedicines-10-01668-s001.zip › Table S5.pdf]

Table S5. Impact of chronic medication on Cox regression analyses of HDL parameters as predictors of 1-year mortality in AHF patients

		HDL-apoA-II (mg/dL)	HDL2-apoA-II (mg/dL)	HDL3-apoA-II (mg/dL)
Univariable	HR (95% CI) per 1 SD	0.50 (0.41-0.62)	0.73 (0.60-0.89)	0.51 (0.40-0.63)
	p-value	**<0.001**	**0.002**	**<0.001**
	events/N	117/314	117/314	117/314
Base	HR (95% CI) per 1 SD	0.67 (0.47-0.94)	0.72 (0.54-0.95)	0.59 (0.43-0.80)
	p-value	**0.020**	**0.019**	**<0.001**
	events/N	111/302	111/302	111/302
Base + furosemide	HR (95% CI) per 1 SD	0.67 (0.47-0.94)	0.72 (0.54-0.95)	0.59 (0.43-0.80)
	p-value	**0.020**	**0.019**	**<0.001**
	events/N	111/302	111/302	111/302
Base + MRA	HR (95% CI) per 1 SD	0.68 (0.48-0.95)	0.73 (0.55-0.96)	0.60 (0.44-0.82)
	p-value	**0.025**	**0.027**	**0.001**
	events/N	111/302	111/302	111/302
Base + beta-blockers	HR (95% CI) per 1 SD	0.70 (0.50-1.00)	0.71 (0.53-0.94)	0.62 (0.46-0.85)
	p-value	**0.048**	**0.017**	**0.003**
	events/N	111/302	111/302	111/302
Base + ACEI	HR (95% CI) per 1 SD	0.66 (0.47-0.93)	0.72 (0.54-0.95)	0.59 (0.43-0.80)
	p-value	**0.018**	**0.020**	**<0.001**
	events/N	111/302	111/302	111/302
Base + statins	HR (95% CI) per 1 SD	0.67 (0.47-0.95)	0.72 (0.54-0.95)	0.59 (0.43-0.81)
	p-value	**0.026**	**0.021**	**<0.001**
	events/N	111/302	111/302	111/302
Base + metformin	HR (95% CI) per 1 SD	0.69 (0.49-0.97)	0.73 (0.55-0.97)	0.59 (0.45-0.90)
	p-value	**0.034**	**0.028**	**<0.001**
	events/N	111/302	111/302	111/302
Base + ASA	HR (95% CI) per 1 SD	0.63 (0.45-0.90)	0.71 (0.54-0.94)	0.57 (0.42-0.78)
	p-value	**0.010**	**0.016**	**<0.001**
	events/N	111/302	111/302	111/302

Significant p-values (<0.05) are depicted in bold.
Base: Adjusted for age, sex, BMI, MAP, eGFR, BUN, CRP, NT-proBNP, hemoglobin, ALT, albumin, and total cholesterol.

ACEI, angiotensinogen converting enzyme inhibitor; AHF, acute heart failure; Ang II, angiotensin II; ASA, acetylsalicylic acid; HDL, high-density lipoprotein; HDL-C, high-density lipoprotein cholesterol; HR, hazard ratio; MRA, mineralocorticoid receptor antagonist; SD, standard deviation.
