# Supplementary material for: HDL-apoA-II Is Strongly Associated with 1-Year Mortality in Acute Heart Failure Patients
Source: Biomedicines. 2022 Jul 11;10(7):1668. doi: 10.3390/biomedicines10071668 (PMC9313377; doi:10.3390/biomedicines10071668)
Supplement: Supplementary file 1 [file biomedicines-10-01668-s001.zip › Table S6.pdf]

Table S6. Multivariable Cox regression analyses of HDL parameters as predictors of 1-year mortality in AHF patients

Variable	Adjusted *			
	SD	HR (95% CI) per 1 SD	p-Value	Events/N
HDL-p (μmol/L)	8.3	0.65 (0.51-0.84)	**0.001**	117/312
SHDL-p (μmol/L)	7.4	0.69 (0.51-0.93)	**0.015**	101/289
HDL-apoA-I (mg/dL)	29.4	0.79 (0.63-0.98)	**0.035**	116/312
HDL1-apoA-I (mg/dL)	16.4	1.07 (0.89-1.28)	0.501	116/312
HDL2-apoA-I (mg/dL)	4.6	0.86 (0.70-1.06)	0.165	116/312
HDL3-apoA-I (mg/dL)	6.1	0.68 (0.54-0.87)	**0.002**	116/312
HDL4-apoA-I (mg/dL)	16.5	0.62 (0.48-0.81)	**<0.001**	116/312

Significant associations are depicted in bold.
* Adjusted for age, sex, MAP, eGFR, BUN, LDL-cholesterol, and NT-proBNP.
apoA-I, apolipoprotein A-I; BUN, blood urea nitrogen; eGFR, estimated glomerular filtration rate; HDL-p, high-density lipoprotein particle concentration; HR, hazard ratio; LDL, low-density lipoprotein; MAP, mean arterial pressure; NT-proBNP, N-terminal pro brain natriuretic peptide; S, small; SD, standard deviation.
