# Supplementary material for: HDL-apoA-II Is Strongly Associated with 1-Year Mortality in Acute Heart Failure Patients
Source: Biomedicines. 2022 Jul 11;10(7):1668. doi: 10.3390/biomedicines10071668 (PMC9313377; doi:10.3390/biomedicines10071668)
Supplement: Supplementary file 1 [file biomedicines-10-01668-s001.zip › Table S7.pdf]

Table S7. Correlation analyses of HDL parameters with laboratory and clinical parameters

		HDL-apoA-I (mg/dL)	HDL-p (µmol/L)	SHDL-p (µmol/L)
Protein (g/L)	r	0.03	0.13	0.17
	p	0.635	0.021	**0.005**
	n	303	303	281
Albumin (g/L)	r	0.32	0.45	0.37
	p	**<0.001**	**<0.001**	**<0.001**
	n	303	303	280
ALT (U/L)	r	-0.02	0.05	0.13
	p	0.733	0.358	0.031
	n	314	314	290
AST (U/L)	r	-0.05	-0.05	0.02
	p	0.332	0.338	0.755
	n	314	314	290
BUN (mmol/L)	r	-0.24	-0.37	-0.34
	p	**<0.001**	**<0.001**	**<0.001**
	n	313	313	290
eGFR	r	0.17	0.25	0.22
(ml/min/1.73m^2)	p	**0.002**	**<0.001**	**<0.001**
	n	314	314	290
CK (U/L)	r	0.07	0.13	0.15
	p	0.213	0.023	**0.010**
	n	314	314	290
hsTnI (U/L)	r	0.02	0.09	0.15
	p	0.704	0.106	**0.014**
	n	311	311	287
NT-proBNP (pg/mL)	r	-0.16	-0.26	-0.26
	p	**0.005**	**<0.001**	**<0.001**
	n	314	314	290
CRP (mg/L)	r	-0.33	-0.32	-0.21
	p	**<0.001**	**<0.001**	**<0.001**
	n	314	314	290
IL-6 (pg/mL)	r	-0.17	-0.22	-0.15
	p	**0.002**	**<0.001**	**0.009**
	n	314	314	290
Hemoglobin (g/L)	r	0.18	0.31	0.33
	p	**0.001**	**<0.001**	**<0.001**
	n	314	314	290
MAP (mm Hg)	r	0.29	0.35	0.29
	p	**<0.001**	**<0.001**	**<0.001**
	n	314	314	290
SPAP (mm Hg)	r	-0.20	-0.28	-0.29
	p	**0.001**	**<0.001**	**<0.001**
	n	259	258	237

Data presented are the Spearman correlation coefficient r, the corresponding p-values, and the number of available samples (n).

P-values of < 0.017 are considered significant after a Bonferroni correction for multiple testing and are depicted in bold.
apoA-I, apolipoprotein A-I; ALT, alanine aminotransferase; AST, aspartate aminotransferase; BUN, blood urea nitrogen; CK, creatin kinase; CRP, C-reactive protein; eGFR, estimated glomerular filtration rate; HDL-p, HDL particle concentrations; hsTnI, high-sensitivity troponin I; IL-6, interleukin-6; MAP, mean arterial pressure; NT-proBNP, N-terminal pro brain natriuretic peptide; S, small; SPAP, systolic pulmonary pressure.
