# Supplementary material for: HDL-apoA-II Is Strongly Associated with 1-Year Mortality in Acute Heart Failure Patients
Source: Biomedicines. 2022 Jul 11;10(7):1668. doi: 10.3390/biomedicines10071668 (PMC9313377; doi:10.3390/biomedicines10071668)
Supplement: Supplementary file 1 [file biomedicines-10-01668-s001.zip › Table S8.pdf]

Table S8. Serum levels of HDL parameters in various groups of AHF patients

		HDL-apoA-I (mg/dL)	HDL-p (µmol/L)	SHDL-p (µmol/L)
CAD	no (n=158)	124.5 (48.0-240.4)	21.9 (4.5-42.7)	15.0 (6.1-37.9)
	yes (n=156)	122.1 (52.4-210.0)	23.9 (5.9-56.3)	19.3 (6.1-40.4)
		p=0.987	p=0.034	**p<0.001**
MetS	no (n=98)	132.3 (73.0-240.4)	25.2 (5.2-56.3)	17.6 (6.1-40.4)
	yes (n=216)	118.1 (48.0-193.1)	21.7 (4.5-42.7)	16.2 (6.1-37.9)
		**p<0.001**	**p=0.002**	p=0.364
AF	no n=(144)	128.8 (48.0-216.5)	25.5 (4.5-56.3)	19.8 (6.5-40.4)
	yes (n=170)	119.9 (60.4-240.4)	20.9 (5.2-41.6)	14.2 (6.1-36.4)
		**p=0.001**	**p<0.001**	**p<0.001**
Venous overload *	no (n=66)	133.6 (90.6-213.1)	29.0 (16.3-42.7)	21.4 (11.4-37.3)
	yes (n=248)	119.7 (48.0-240.4)	21.5 (4.5-56.3)	15.0 (6.1-40.4)
		**p<0.001**	**p<0.001**	**p<0.001**
AHF type	New onset AHF (n=27)	139.4 (94.3-182.6)	32.5 (12.3-42.7)	24.3 (16.3-37.9)
	AHF following CHF (n=287)	122.4 (48.0-240.4)	22.2 (4.5-56.3)	16.1 (6.1-40.4)
		**p<0.001**	**p<0.001**	**p<0.001**

Data are presented as median and minimum to maximum. Differences between the groups were tested with the Mann-Whitney U test.
P-values < 0.017 are considered significant after a Bonferroni correction for multiple testing and are depicted in bold.
AF, atrial fibrillation; AHF, acute heart failure; apoA-I, apolipoprotein A-I; CAD, coronary artery disease; CHF, chronic heart failure; HDL, high-density lipoprotein; HDL-p, HDL particle concentration; MetS, metabolic syndrome; S, small.
*Any of the following: enlarged liver, peripheral edema, ascites or jugular venous distension.
